# Genotypes Associated with *Listeria monocytogenes* Isolates Displaying Impaired or Enhanced Tolerances to Cold, Salt, Acid, or Desiccation Stress

**DOI:** 10.3389/fmicb.2017.00369

**Published:** 2017-03-08

**Authors:** Patricia Hingston, Jessica Chen, Bhavjinder K. Dhillon, Chad Laing, Claire Bertelli, Victor Gannon, Taurai Tasara, Kevin Allen, Fiona S. L. Brinkman, Lisbeth Truelstrup Hansen, Siyun Wang

**Affiliations:** ^1^Department of Food, Nutrition, and Health, University of British ColumbiaVancouver, BC, Canada; ^2^Department of Molecular Biology and Biochemistry, Simon Fraser UniversityBurnaby, BC, Canada; ^3^Laboratory for Foodborne Zoonoses, Public Health Agency of CanadaLethbridge, AB, Canada; ^4^Institute for Food Safety and Hygiene, University of ZurichZurich, Switzerland; ^5^Division for Microbiology and Production, National Food Institute, Technical University of DenmarkKongens Lyngby, Denmark

**Keywords:** *Listeria monocytogenes*, stress tolerance, whole genome sequencing, sequence typing, food safety, plasmids, internalin A

## Abstract

The human pathogen *Listeria monocytogenes* is a large concern in the food industry where its continuous detection in food products has caused a string of recalls in North America and Europe. Most recognized for its ability to grow in foods during refrigerated storage, *L. monocytogenes* can also tolerate several other food-related stresses with some strains possessing higher levels of tolerances than others. The objective of this study was to use a combination of phenotypic analyses and whole genome sequencing to elucidate potential relationships between *L. monocytogenes* genotypes and food-related stress tolerance phenotypes. To accomplish this, 166 *L. monocytogenes* isolates were sequenced and evaluated for their ability to grow in cold (4°C), salt (6% NaCl, 25°C), and acid (pH 5, 25°C) stress conditions as well as survive desiccation (33% RH, 20°C). The results revealed that the stress tolerance of *L. monocytogenes* is associated with serotype, clonal complex (CC), full length *inlA* profiles, and the presence of a plasmid which was identified in 55% of isolates. Isolates with full length *inlA* exhibited significantly (*p* < 0.001) enhanced cold tolerance relative to those harboring a premature stop codon (PMSC) in this gene. Similarly, isolates possessing a plasmid demonstrated significantly (*p* = 0.013) enhanced acid tolerance. We also identified nine new *L. monocytogenes* sequence types, a new *inlA* PMSC, and several connections between CCs and the presence/absence or variations of specific genetic elements. A whole genome single-nucleotide-variants phylogeny revealed sporadic distribution of tolerant isolates and closely related sensitive and tolerant isolates, highlighting that minor genetic differences can influence the stress tolerance of *L. monocytogenes*. Specifically, a number of cold and desiccation sensitive isolates contained PMSCs in σ^B^ regulator genes (*rsbS, rsbU, rsbV*). Collectively, the results suggest that knowing the sequence type of an isolate in addition to screening for the presence of full-length *inlA* and a plasmid, could help food processors and food agency investigators determine why certain isolates might be persisting in a food processing environment. Additionally, increased sequencing of *L. monocytogenes* isolates in combination with stress tolerance profiling, will enhance the ability to identify genetic elements associated with higher risk strains.

## Introduction

*Listeria monocytogenes* is a ubiquitous bacterial foodborne pathogen that is most recognized for its ability to grow at temperatures as low as −0.4°C (Walker et al., 1990) and cause listeriosis, a serious disease with an average mortality rate of 30% among at-risk people (Yildiz et al., 2007). In addition to possessing cold tolerance, *L. monocytogenes* is also capable of surviving many other food-related stresses including high osmolarity (Shabala et al., 2008) and low pH (Sorrells et al., 1989), further adding to its hardiness. Additionally, cross contamination of foods is facilitated by biofilm formation (Chavant et al., 2002; Møretrø and Langsrud, 2004; Moltz, 2005; Di Bonaventura et al., 2008; Hingston et al., 2013), and the ability of the organism to survive desiccation for extended periods of time on food contact surfaces (Vogel et al., 2010). Post-processing levels of *L. monocytogenes* contamination in foods are usually low (Fenlon et al., 1996; Kozak et al., 1996; Cabedo et al., 2008) and unlikely to cause disease (Buchanan et al., 1997; Chen et al., 2003). It is therefore, refrigerated, ready-to-eat (RTE) foods with extended shelf lives and the potential for regrowth that present the largest risk to consumers.

Both Canada and the EU have adopted regulations for the control of *L. monocytogenes* in RTE foods (Health Canada, 2011; Luber, 2011), allowing up to 100 CFU/g in foods that do not permit growth beyond this level within the shelf-life of the product, and a zero tolerance policy for foods identified as supporting growth. When validating growth inhibition of *L. monocytogenes* in stabilized RTE foods, it is important that the strains used represent the extremes of *L. monocytogenes'* stress response behavior. In the US, the zero tolerance policy is applicable for all food products (US FDA, 2016). However, nationwide outbreaks continue to occur in the US. To date, there have been three multistate listeriosis outbreaks in 2016 that were associated with frozen vegetables, packaged salads, and raw milk and resulted in 29 illnesses and 4 deaths (CDC, 2016a). These numbers are yet to exceed that of 2015 where two multistate outbreaks involving soft cheese and ice cream resulted in 40 illnesses and 6 deaths (CDC, 2016a).

In 2013, the US established the *Listeria* Whole Genome Sequencing (WGS) Project to assist in detecting, investigating, and mitigating foodborne outbreaks (CDC, 2016b). Though valuable for tracing outbreaks, WGS is not routinely used to determine the stress tolerance of outbreak strains. However, WGS provides the information that could potentially lead to identification of molecular biomarkers related to the stress tolerance of *L. monocytogenes* isolates. Such biomarkers could greatly aid in monitoring the risks of *L. monocytogenes* contamination and regrowth in food products and processing environments (Jacquet et al., 2004).

Currently, one common molecular biomarker used for *L. monocytogenes'* virulence is the internalin A encoding gene (*inlA*) which can contain one of several different premature stop codons producing truncated and secreted proteins associated with attenuated virulence (Jonquieres et al., 1998; Jacquet et al., 2004; Rousseaux et al., 2004; Nightingale et al., 2005; Felicio et al., 2007; Handa-Miya et al., 2007; Roldgaard et al., 2009; Van Stelten et al., 2011). Recently, Kovacevic et al. (2013) discovered that full-length variants of *inlA* were more prevalent among fast cold-adapting *L. monocytogenes* strains than intermediate and slow cold-adapting strains, suggesting that *inlA* profiling may also be suitable for predicting the cold tolerance of strains. Another potential biomarker is the *L. monocytogenes* stress survival islet 1 (SSI-1). Included in this five gene cluster (*lmo0444–lmo0448*) are two genes (*gadT1* and *gadD1*) from the glutamate decarboxylase acid resistance system which has been shown to significantly improve the growth of *L. monocytogenes* in mildly acidic environments (Cotter et al., 2005). Additionally, an *L. monocytogenes* SSI-1 deletion mutant exhibited impaired growth at low pH (pH 4.8), high salt (7.5% NaCl), and on frankfurters stored at 4°C (Ryan et al., 2010). Further, research with naturally occurring SSI-1 positive and negative strains is needed to determine if this island would be a suitable biomarker for predicting stress tolerance phenotypes.

To date, studies which evaluated the stress tolerances of *L. monocytogenes* isolates have focused on associating phenotypes with genetic lineages (Bergholz et al., 2010), serotypes (Junttila et al., 1988; Barbosa et al., 1994; Ribeiro et al., 2014), and isolation sources (Begot et al., 1997; Durack et al., 2013). However, few significant differences between these groups were observed, suggesting that the diversity among isolates within these means of classification is not definitive for predicting phenotypic behavior. Instead, stronger phenotype associations might be observed among more closely related isolates, e.g., those sharing the same sequence type (ST) or clonal complex (CC). Additionally, the presence of specific genetic elements (e.g., *inlA* and SSI-1) may also influence the stress tolerance phenotypes of isolates as well as more minor genetic differences such as single nucleotide variants (SNVs).

The objective of this study was to use a combination of phenotypic analyses and WGS to elucidate novel associations between *L. monocytogenes* genotypes and food-related stress tolerance phenotypes with the goal of identifying biomarkers that can be used to predict the stress tolerances of food-chain isolates. To accomplish this, 166 *L. monocytogenes* isolates were evaluated on their ability to grow in cold (4°C), salt (6% NaCl), and acid (pH 5) stress conditions as well as survive desiccation stress (33% RH). Factors investigated for potential associations with the observed phenotypes were: genetic lineage, serotype, CC, *inlA* profiles, and the presence of a plasmid, SSI-1, unique SNVs, and *Listeria* genomic island 1 (LGI1).

## Materials and methods

### Isolates and culture conditions

A collection of 166 *Listeria monocytogenes* isolates from Canada and Switzerland were used in this collaborative study. This included: (i) 159 food and food processing environment isolates from Canada (*n* = 139) and Switzerland (*n* = 20), (ii) six isolates from sporadic human listeriosis cases in Switzerland, and (iii) one isolate from an asymptomatic human (Table S1). All human isolates were anonymized and no ethical approval was required as per the institutional and national guidelines. Isolates were stored at −70°C in brain heart infusion broth (BHIB, Difco, Fisher Scientific, Canada) +20% glycerol and routinely cultured at 30°C on BHI agar (BHIA, Difco, Fisher Scientific) plates.

### Whole genome sequencing

Genomic DNA was isolated using the PureLink Mini Kit from Life Technologies, Canada. PicoGreen quantification was performed (Invitrogen, Canada) and DNA was assessed using the NanoDrop 2000 (Fisher Scientific). Genomic DNA samples of sufficient quality and quantity were sequenced by Genome Quebec (Montréal, QC, Canada) using TruSeq automated library preparation (Illumina) and paired-end, 100 bp sequencing on the Illumina Hi-Seq. Between 4.9 and 16.5 million high quality reads remained after quality control for each genomic library. Raw FASTQ files were trimmed using Cutadapt in Trim Galore! version 0.4.1 and *de novo* genome assembly was performed using SPAdes version 3.1.0 (careful option used; Bankevich et al., 2012). Low coverage (<10) and small contigs (<200 bp) were removed from assemblies using a custom perl script. Assemblies were subsequently annotated using Prokka version 1.5.2 (genus *Listeria*, species *monocytogenes*; Seemann, 2014). Assembled sequences were deposited into the NCBI Whole Genome Shotgun (WGS) database under Bioproject PRJNA329415.

### Lineage determination

To classify isolates into genetic lineages, a reference free, k-mer based single nucleotide variants (SNV) phylogeny was generated using the kSNP 3.0 program (Gardner et al., 2015) and reference isolates for the major lineages of *L. monocytogenes* (LI—F2365; LII—EGD-e; LIII—HCC23; LIV—J1-208). The resulting maximum parsimony tree (based on the consensus of 100 trees) clearly segregated the four lineages.

### Multi locus sequence typing

To group isolates based on their epidemiological context, *in silico* MLST was performed using the Center for Genomic Epidemiology's MLST typing tool (https://cge.cbs.dtu.dk/services/MLST/). Clonal Complexes (CCs) were assigned based on the Pasteur Institute schema (http://www.pasteur.fr/recherche/genopole/PF8/mlst/Lmono.html). Novel sequence types (STs) were confirmed using Sanger sequencing and submitted to the Pasteur Institute Database for new assignments (http://bigsdb.pasteur.fr/listeria/listeria.html).

### *In silico* serogroup/serotype assignment

Antibody-based serotyping was conducted on a subset of isolates (*n* = 91) within both the current study and previous studies (Arguedas-Villa et al., 2010; Kovacevic et al., 2013). Remaining isolates were assigned one or more possible serotypes by performing a MegaBLAST search (>95% nt identity) ncbi-blast+ v. 2.3.0 available at: ftp://ftp.ncbi.nlm.nih.gov/blast/executables/blast+/LATEST/) for four genes used in a multiplex PCR developed by Doumith et al. (2004). Additionally, predictive serotypes were assigned to isolates with STs that are known to be associated with a specific serotype.

### Targeted genomic element screenings

The genes and genomic regions evaluated in this study were (1) the plasmid replicon gene *repA*, used to indicate the presence of a plasmid (Kuenne et al., 2010), (2) *emrE*, representing *Listeria* genomic island 1 (LGI1), a 50 kb island with putative roles in stress tolerance and persistence (Gilmour et al., 2010), and (3) stress survival islet 1 (SSI-1), a five gene cluster previously identified as having a role in *L. monocytogenes'* response to cold, osmotic, and acid stress conditions (Ryan et al., 2010). Additionally, the coding sequence of *inlA* was investigated to determine if isolates possessed a full length sequence or a premature stop codon (PMSC) mutation. *emrE*, SSI-1, and *inlA* were screened for among isolate sequence assemblies using MegaBLAST (>95% nt identity) and *repA* was screened for using BLASTP (>30% aa identity over >80% coverage). *inlA* and *repA* sequences were then extracted from the isolate assemblies for further analysis.

### Identification of putative plasmid contigs

Detection of *repA* sequences meant that at least one contig belonged to a putative plasmid. To identify additional plasmid associated contigs, isolate assemblies were aligned to the closed genome of *L. monocytogenes* EDG-e (Accession: NC_003210.1) using Contig Mover in Mauve version 2.3.1 (Rissman et al., 2009). Contigs not aligning to the EDG-e chromosome were compared to published *L. monocytogenes* plasmids (Kuenne et al., 2010) by BLAST. Contigs were excluded if they displayed open readings frames associated with chromosomal DNA (e.g., rRNA, tRNA) or did not align to any of the *Listeria* associated plasmids annotated by Kuenne et al. (2010): pLM33, pLM1-2bUG1, pLM5578, pLM80, and pLI100. A summary of the putative plasmid contigs found within each isolate can be found in Table S1.

### Cold tolerance assay

Overnight cultures grown in BHIB at 37°C were standardized to 10^9^ CFU/ml using spectrophotometric methods, and diluted in pre-chilled BHIB to yield a final density of 10^3^ CFU/ml and stored at 4°C (previously described in Arguedas-Villa et al., 2010). The bacterial density was enumerated daily for the first 4 days and then bi-weekly for up to 5 weeks by plating on tryptic soy agar (BD, Fisher Scientific) +6% yeast extract (BD, Fisher Scientific). The resulting growth curves were fitted using a four parameter logistic model described by Dalgaard and Koutsoumanis (2001).

### Salt and acid tolerance assay

Isolates were assessed for salt and acid tolerance using modified versions of published protocols (Cotter et al., 2005; Van Der Veen et al., 2008; Bergholz et al., 2010). In short, overnight cultures grown in BHIB at 30°C were diluted in either BHIB+6% (w/w) NaCl or BHIB adjusted to pH 5 (with 1 M HCl) to achieve a final concentration of 10^7^ CFU/ml. From these cultures, 200 μl was added in duplicate (technical replicates) to 96-well-plates (Costar™ clear polystyrene, Fisher Scientific) that were incubated at 25°C in a microplate reader (Spectramax, V6.3; Molecular Devices, Sunnyvale, CA). A temperature of 25°C was used to assess isolate salt and acid tolerance under non-intracellular or cold stress conditions. The absorbance (A_600nm_) of each well was recorded every 30 min until all isolates reached stationary phase (~26 h) and the resulting growth curves were fitted to the Baranyi and Roberts model (Baranyi and Roberts, 1994) using DMfit (v3.5) available on the ComBase browser (http://browser.combase.cc/DMFit.aspx).

### Desiccation tolerance assay

Cultures grown for 24 h in BHIB at 20°C were diluted to 10^7^ CFU/ml in buffered peptone water (BD, Fisher Scientific) and 10 μl (10^5^ CFU) was spotted in duplicate (technical replicates) on the bottom of wells in lid-less 96-well-plates. The plates were then stored for 3 days at 20°C in desiccators (SICCO, Bohlender, Germany) pre-conditioned to 33% relative humidity (RH) using a saturated solution of MgCl_2_ (protocol adapted from Hingston et al., 2015). The RH of the chambers was monitored throughout the desiccation periods using data loggers (included with desiccators). A temperature of 20°C was used to simulate desiccation conditions that might occur in a food plant. Following desiccation, the plates were rehydrated with 200 μl of BHIB, and incubated at 25°C in a plate reader where the A_600nm_ of each well was recorded every 30 min until all isolates reached stationary phase (~24 h). The resulting growth curves were then fitted to the Baranyi and Roberts model and the model parameters recorded.

### Phenotype designations and statistical analyses

For all four stress exposure experiments, a minimum of two biological replicates with two technical replicates each, were conducted for all isolates. Based on the findings of Aryani et al. (2015), the data was standardized for biological variability between replicates by dividing isolate growth parameters by the median value for each experimental run, thereby making the median equal to 1. The median was selected for standardization rather than the mean to avoid the influence of very stress sensitive isolates. Model parameters (LPD, lag phase duration; μ_max_, maximum growth rate; N_max_, maximum cell density) were averaged across biological replicates and presented as standardized (std) values. For isolates where the average std values had a standard deviation (*SD*) >0.05, additional replicates were completed to obtain more representative means. Isolates were considered tolerant or sensitive to cold, salt, or acid stress if they had an average std-μ_max_ > or < than 1 *SD* from the median, respectively. All remaining isolates were considered to have intermediate stress tolerance. For desiccation stress survival the model parameter of most interest was the LPD, indicating the time to (detectable) regrowth (TRG) that is negatively correlated with the number of cells, which survived the desiccation treatment. Isolates were classified as desiccation tolerant or sensitive if they had an average std-TRG < or > than 1 *SD* from the median, respectively. A standard curve generated using five cell levels (10^1^–10^5^ CFU) produced a correlation of *y* = −0.25x + 2.07 (*R*^2^ = 0.97) where y is the TDR and x is the log_10_ number of viable cells in each well following desiccation.

To elucidate potential associations between the factors we investigated, statistical tests were performed using IBM SPSS Statistics version 23. Specifically, individual two-tailed *T*-tests and one-way ANOVAs with Tukey *post-hoc* tests were used to compare the average standardized stress tolerance model parameters of two (±plasmid, ±SSI-1, ±LGI1, lineage I and II, *repA* group 1 vs. group 2 isolates) or more groups (serotypes, CCs, *inlA* profiles, and sensitive, intermediate and stress tolerant groups), respectively. G^*^Power 3.1.9.2 (Faul et al., 2007, 2009) was used to determine the minimum sample sizes required to ensure a power of 0.80 for all statistical tests. All data sets were accessed for outliers, homogeneity of variances (Levene's test), and normality (Shapiro-Wilk's test). Where homogeneity of variances could not be achieved, Welch *T*-tests and Welch ANOVAs in combination with Games-Howell *post-hoc* tests were used. *P*-values below 0.05 were considered significant for all comparisons.

### Phylogenetic reconstruction based on core genome single nucleotide variants

Parsnp, a tool within Harvest suite of tools (Treangen et al., 2014), was used to perform core genome alignment of all 166 *de novo* assembled genomes and the reference *L. monocytogenes* EGD-e strain in order to identify single nucleotide variants (SNVs) within the core genome. SNVs clustered within 20 base pairs were removed as these may indicate repetitive regions containing more erroneous SNV calls. The remaining high quality SNVs were used to generate maximum likelihood trees using the RaxML version 8 (Stamatakis, 2014) on the CIPRES science gateway (Miller et al., 2010) using default parameters (including the GTRCAT nucleotide model and 100 bootstrap replicates). Corresponding heatmaps containing additional genotype and phenotype information were generated in R version 2.15.1 (Team, 2016) using the heatmap.2 function from the gplots library.

### SNV detection

SNVs were also detected against the *Listeria monocytogenes* EGD-e (NC_003210.1) reference genome. SMALT version 0.7.6 (http://www.sanger.ac.uk/science/tools/smalt-0) with default parameters except “–i 330” was used to first align raw reads against the reference. Samtools version 1.2 (Li, 2011) was used on these assemblies to sort the aligned reads (“samtools sort”), remove potential PCR duplicates (“samtools rmdup”) and call the SNVs (“samtools mpileup”). Additional filtering of SNV calls included removing those with a read depth <50 and heterozygous genotypes (since our genomes are haploid) using the “bcftools filter” command. SNVs found in repetitive regions of the genome as assessed by the index of repetitiveness (Schwender et al., 2004) were also removed manually. The remaining high confidence SNVs were annotated using SNPEff version 4.1 (Cingolani et al., 2012) with the *Listeria*_monocytogenes_EGD_e_uid61583 annotation. Synonymous SNVs were also removed in the end for identification of non-synonymous or potential regulatory SNVs that may be contributing to phenotypic differences in cold growth.

### Statistical methods for elucidating SNVs associated with stress tolerance phenotypes

SNPSift version 4.1 “CaseControl” (Cingolani et al., 2012) was used to run a Fisher Exact test to identify SNVs that were significantly associated with case vs. control groups. To identify SNVs only found in tolerant isolates, these were used as the case group, while all others were used as the control group. Since this did not yield any results, subsequently, the sensitive isolates alone were used as the control group so as to allow SNVs to be seen in intermediate growers. This method has certain limitations in that certain associations may require very large sample sizes to become statistically significant, especially considering genetic heterogeneity leads to the same phenotype or the potential for multiple SNVs to interact. An alternative approach, Random Forests™ (Breiman, 2001), was also used to discover important SNVs in distinguishing stress tolerant and sensitive groups since previous genome wide association studies have shown random forests outperform the Fisher Exact test in these special cases (Lunetta et al., 2004; Schwender et al., 2004; Bureau et al., 2005; De Lobel et al., 2010; Bulinski et al., 2011). The RandomForest™ version 4.6–10 library was used in R with default parameters except “importance = TRUE, proximity = TRUE, ntree = 5000.” This allows the method to run as a classifier that then ranks SNVs on their ability to classify isolates based on their phenotypic designation.

### Genomic islands analysis

Annotated draft genomes were submitted to IslandViewer 3 (Dhillon et al., 2015) using *L. monocytogenes* EGD-e (NC_003210.1) as a reference for contig reordering. Genomic islands were predicted using IslandPath-DIMOB (Hsiao et al., 2005) and SIGI-HMM (Waack et al., 2006). Predicted genomic islands positioned on the genome within <10 kb of each other were merged into one single region.

To form groups of similar genomic islands, the genetic distance between genomic island sequences was computing using Mash (parameter –s 2000; Ondov et al., 2016) and groups of similar sequences were identified using hclust and cutree in R.

## Results

### Genetic characteristics of *L. monocytogenes* isolates based on WGS data

The complete sequenced genome assembly sizes of the isolates ranged from 2.56 to 3.13 Mbp with a mean size of 2.97 Mbp (Table S1). Isolates belonged to one of three different lineages: LI (*n* = 44, serotypes 4b, 1/2b, 3b, and 3c), LII (*n* = 121, serotypes 1/2a, 1/2c, and 3a), and LIII (*n* = 1, serotype 4c). The majority of isolates were serotype 1/2a (*n* = 92), followed by 1/2c and 4b (*n* = 25 each), 1/2b (*n* = 18), 3a (*n* = 2), and 3b and 4c (*n* = 1 each; Table 1). The exact serotype was not determined for two remaining isolates. Beyond serotypes, our isolates belonged to 36 different known STs and a further nine were assigned novel STs (ST1017-1025). Isolates also belonged to one of 29 different CCs with a further seven isolates being unique non-clonal singletons. The most prevalent CCs in the collection were CCs 9, 8, and 7 (Table 2). Other less common CCs in decreasing prevalence included CCs 11, 155, 1, 3, and 321 (Table 2). Interestingly, only one CC121 isolate existed in our collection. This is surprising given that CC121 is often highly prevalent among *L. monocytogenes* food-associated isolates (Parisi et al., 2010; Chenal-Francisque et al., 2011; Martín et al., 2014; Ebner et al., 2015; Maury et al., 2016).

**Table 1:** **Genetic characteristics and prevalence of sensitive and tolerant phenotypes among *L. monocytogenes* belonging to different serotypes**.

**Serotype**	***n*** **(%)**	**Plasmid+ (%)^a^**	**Full length *inlA* (%)^a^**	**SSI-1+ (%)^a^**	**CS (%)^a^**	**CT (%)^a^**	**SS (%)^a^**	**ST (%)^a^**	**AS (%)^a^**	**AT (%)^a^**	**DS (%)^a^**	**DT (%)^a^**
4b	25 (15)	4 (16)	14 (56)	4 (16)	4 (16)	2 (8)	1 (4)	3 (12)	1 (4)	4 (16)	2 (8)	3 (12)
1/2b	18 (11)	14 (78)	15 (83)	17 (94)	0	2 (11)	3 (17)	2 (11)	0	7 (39)	2 (11)	4 (22)
1/2a	92 (55)	49 (53)	85 (92)	65 (71)	4 (4)	11 (12)	18 (20)	12 (13)	22 (24)	7 (8)	10 (11)	11 (12)
1/2c	25 (15)	21 (84)	3 (12)	25 (100)	5 (20)	3 (12)	4 (16)	0	2 (8)	4 (16)	4 (16)	4 (16)
3a	2 (1)	2 (100)	0	2 (100)	0	0	1	0	1	0	0	0
3b	1	1	1	1	0	0	0	0	0	0	0	1
4c	1	0	0	0	0	0	0	0	0	0	1	0
1/2b, 3b, 7	1	0	1	1	0	0	0	0	0	0	0	0
1/2a, 3a	1	1	0	1	0	0	1	0	0	0	1	0
Sum	166	92	119	116	13	18	27	17	26	22	20	23

*CS, cold sensitive; CT, cold tolerant; SS, salt sensitive; ST, salt tolerant; AS, acid sensitive; AT, acid tolerant; DS, desiccation sensitive; DT, desiccation tolerant*.

a *Percentages relate to prevalence within the serotype*.

**Table 2:** **Genetic characteristics and prevalence of sensitive and tolerant phenotypes among *L. monocytogenes* belonging to different clonal complexes**.

**CC**	**Associated serotypes**	***n*** **(%)**	**Plasmid+ (%)^a^**	**Full length *inlA* (%)^a^**	**SSI-1+ (%)^a^**	**CS (%)^a^**	**CT (%)^a^**	**SS (%)^a^**	**ST (%)^a^**	**AS (%)^a^**	**AT (%)^a^**	**DS (%)^a^**	**DT (%)^a^**
1	4b	6 (4)	0	6 (100)	0	2 (33)	0	1 (17)	0	0	1 (17)	1 (17)	0
2	4b	3 (2)	0	3 (100)	0	1 (33)	0	0	2 (67)	0	0	0	0
3	1/2b	6 (4)	5 (83)	6 (100)	6 (100)	0	0	1 (17)	0	0	1 (17)	1 (17)	1 (17)
4	4b	3 (2)	0	3 (100)	0	0	1 (33)	0	0	0	2 (67)	1 (33)	0
5	1/2b	7 (4)	7 (100)	4 (57)	7 (100)	0	1 (14)	0	1 (14)	0	4 (57)	1 (14)	1 (14)
6	4b	7 (4)	4 (57)	0	0	1 (14)	0	0	0	1 (14)	0	0	2 (29)
7	1/2a	17 (10)	10 (59)	17 (100)	17 (100)	2 (12)	1 (6)	8 (47)	0	5 (29)	0	1 (6)	3 (18)
8	1/2a	22 (13)	14 (64)	22 (100)	22 (100)	0	3 (14)	2 (9)	1 (5)	1 (5)	3 (14)	2 (9)	0
9	1/2c, 1/2a	27 (16)	23 (85)	4 (15)	27 (100)	5 (19)	4 (15)	6 (22)	0	3 (11)	4 (15)	6 (22)	4 (15)
11	1/2a	11 (7)	10 (91)	11 (100)	0	0	1 (9)	0	4 (36)	0	1 (9)	0	4 (36)
14	1/2a	3 (2)	0	3 (100)	0	0	1 (33)	0	1 (33)	0	0	0	0
20	1/2a	3 (2)	0	3 (100)	0	0	1 (33)	1 (33)	1 (33)	2 (67)	0	0	0
155	1/2a	8 (5)	1 (13)	8 (100)	8 (100)	1 (13)	0	2 (25)	1 (13)	3 (38)	1 (13)	1 (13)	0
224	1/2b	4 (2)	0	4 (100)	4 (100)	0	1 (25)	2 (50)	0	0	1 (25)	0	3 (75)
315	4b	4 (2)	0	0	4 (100)	0	0	0	0	0	0	0	1 (25)
321	1/2a, 3a	6 (4)	6 (100)	0	6 (100)	0	0	0	1 (17)	5 (83)	0	0	2 (33)
Other	1/2a, 1/2b, 4b, 4c	29 (17)	12 (41)	25 (86)	15 (52)	1 (3)	4 (14)	4 (14)	5 (17)	6 (20)	4 (14)	6 (20)	2 (7)
Sum		166	92	119	116	13	18	27	17	26	22	20	23

*CS, cold sensitive; CT, cold tolerant; SS, salt sensitive; ST, salt tolerant; AS, acid sensitive; AT, acid tolerant; DS, desiccation sensitive; DT, desiccation tolerant*.

a *Percentages relate to prevalence within CCs*.

The plasmid replication gene, *repA*, was observed in 55% (*n* = 92) of our isolates, with a prevalence of 41 and 61% among LI and LII isolates, respectively. Notably, one isolate was observed to contain two putative plasmids as indicated by the presence of two different *repA* containing contigs of 61 and 69 kb. Among serotypes, *repA* was present in 100% of 3a isolates, 84% of 1/2c isolates, 78% of 1/2b isolates, 53% of 1/2a isolates, and 16% of 4b isolates (Table 1). Among CCs, plasmids were observed in >80% of CC 3, 5, 9, 11, and 321 isolates (Table 2).

A phylogeny, constructed on *repA* sequences as described in Kuenne et al. (2010), divided the sequences into two groups. Group 1 included isolates from serotypes 1/2a, 1/2b, 1/2c, and 4b with estimated plasmid sizes ranging from 26 to 88 kb. Group 2 included 1/2a, 1/2b, 1/2c, and 3a serotype isolates, harboring significantly larger plasmids (*p* < 0.0005, 55–100 kb) than those from group 1. These sizes are in line with those observed in Kuenne et al. (2010), supporting the assertion that these contigs belong to plasmids. The most prevalent plasmid size (56553–56554 bp) was observed for 26 isolates from seven different CCs and from both lineages I and II. Also noteworthy is that isolates from Switzerland and Canada contained plasmids with 100% nucleotide identity.

Premature stop codons (PMSCs) in *inlA* were observed in 20% of our isolates encompassing seven (Table S1) of 19 published PMSCs (Jonquieres et al., 1998; Olier et al., 2002, 2003; Rousseaux et al., 2004; Nightingale et al., 2005, 2008; Orsi et al., 2007; Van Stelten and Nightingale, 2008; Van Stelten et al., 2010; Wu et al., 2016) and one novel PMSC at 760aa, which was identified in two serotype 1/2c isolates. The most common PMSC occurred at 9aa (*n* = 13) and was associated with CC9, serotype 1/2c isolates. Ten of the 14 remaining CC9 isolates also had one of four *inlA* PMSCs (326, 576, 685, 760aa) and all CC321 isolates contained *inlA* PMSC's at 700aa (Table S1). An additional 13 isolates, all from the serotype 4b CCs 6 and 315, contained a three codon deletion mutation previously reported in Kovacevic et al. (2013). With the exception of CCs 5 and 9, all isolates from the same CC either contained full length *inlA* or a truncated version.

During the screening of the whole genome sequences, the absence of *lmo1078* was noted among serotype 4b isolates. This gene, which encodes a UDP-glucose pyrophosphorylase, has been previously demonstrated to have a role in *L. monocytogenes* cold growth (Chassaing and Auvray, 2007). It was also observed that 70% (*n* = 116) of strains possessed SSI-1 with this island being most prevalent among serotype 1/2c isolates (100%) followed by 1/2b (94%), 1/2a (71%), and 4b (16%; Table 2). Furthermore, all isolates from CCs 3, 5, 7, 8, 9, 155, 224, 315, and 321 contained SSI-1 (Table 2). All remaining isolates possessed a homolog to F2365_0481 in place of SSI-1 (Ryan et al., 2010), with the exception of the CC121 isolate which possessed *lin0464* and *lin0465* homologs as reported in Hein et al. (2011).

The LGI1 indicator gene, *emrE*, was found in 16 of our isolates and as previously reported (Gilmour et al., 2010; Althaus et al., 2014; Kovacevic et al., 2015), all originated from Canada and 14 were serotype 1/2a ST120-CC8. The remaining two isolates represented novel STs (ST1022 and 1025) that also belonged to CC8. All *emrE* containing isolates also harbored SSI-1 and full length *inlA*.

### Stress tolerance distributions among *L. monocytogenes* isolates

**Figure 1:**
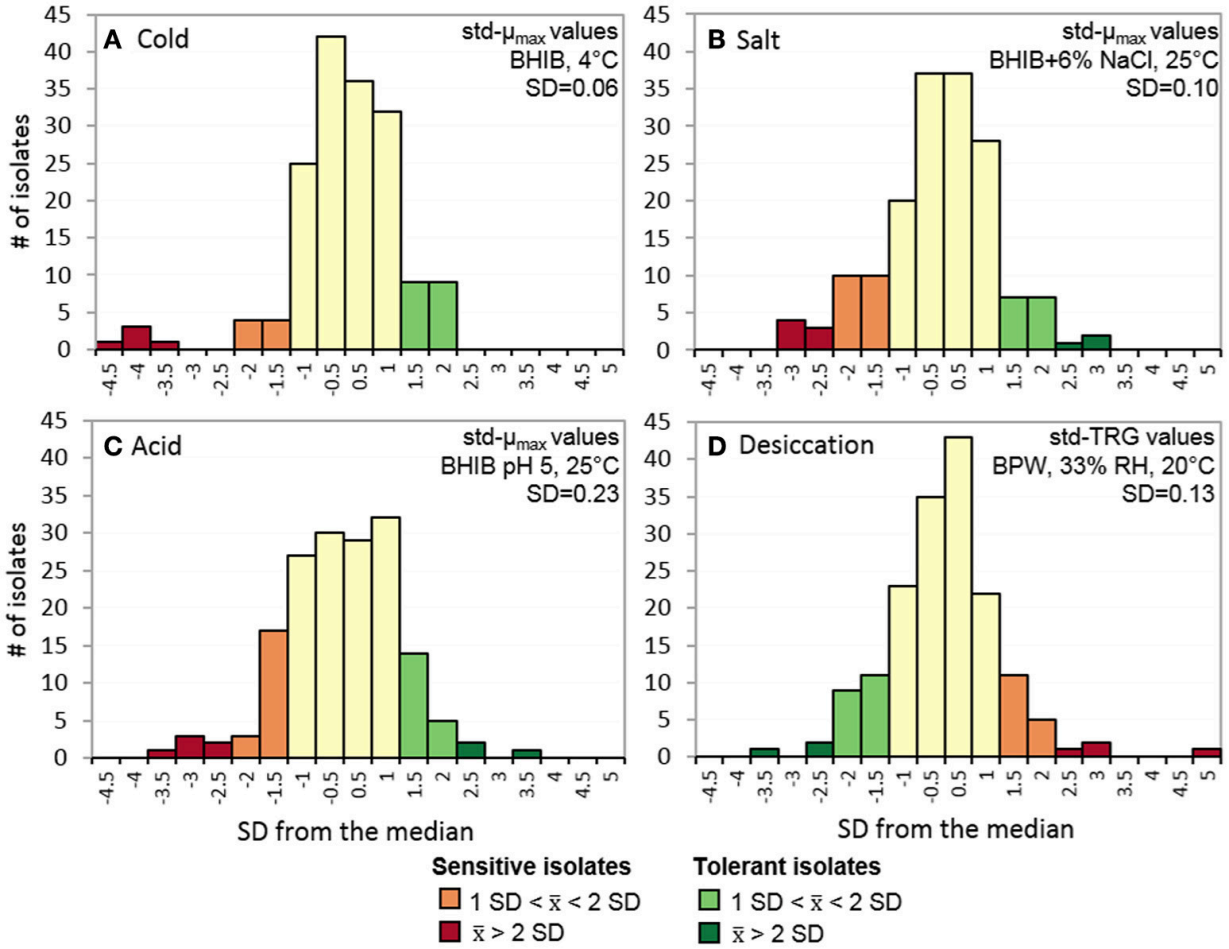
**Stress tolerance distributions of 166 *L. monocytogenes* isolates**. Std-μ_max_ of isolates grown in **(A)** BHIB at 4°C, **(B)** BHIB+6% NaCl at 25°C, and **(C)** BHIB pH 5 at 25°C. **(D)** std-TRG of isolates after being desiccated at 33% RH for 3 days in BPW at 20°C and then rehydrated with BHIB and grown at 30°C. Isolates were classified as sensitive or tolerant if they displayed an average std-μ_max_orstd-TRG >1 *SD* from the median (= 1). std-μ_max_, standardized maximum growth rate; std-TRG, standardized time to detectable regrowth; BHIB, brain heart infusion broth; BPW, buffered peptone water.

All *L. monocytogenes* isolates were evaluated on their ability to grow in cold (4°C), salt (6% NaCl), and acid (pH 5) stress conditions as well as survive desiccation stress (33% RH). The cold growth plate count data was modeled using the Dalgaard and Koutsoumanis (2001) logistic model because it was more accommodating of fewer sampling points [average *R*^2^ = 0.998, mean standard error (MSE) = 0.129]. From the std-μ_max_ values, 13 isolates were classified as cold sensitive and 18 were classified as cold tolerant with average std-μ_max_ values of 0.85 ± 0.08 and 1.09 ± 0.02, respectively (Figure 1A). For the salt, acid, and desiccation tolerance assays, the Baranyi and Roberts (1994) was suitable for modeling the spectrophotometrically obtained data with average *R*^2^ and MSE-values ranging from 0.997 to 0.998 and 0.003 to 0.017, respectively. Overall, 27 and 17 isolates were classified as salt sensitive and tolerant with average std-μ_max_ values of 0.83 ± 0.05 and 1.16 ± 0.05 (Figure 1B); 26 and 22 isolates were classified as acid sensitive and tolerant with average std-μ_max_ values of 0.64 ± 0.14 and 1.34 ± 0.12 (Figure 1C); and 20 and 23 isolates were identified as desiccation sensitive and tolerant isolates with average std-TRGs of 0.81 ± 0.06 and 1.22 ± 0.11 (Figure 1D), respectively.

### Overlapping stress-tolerance phenotypes

**Figure 2:**
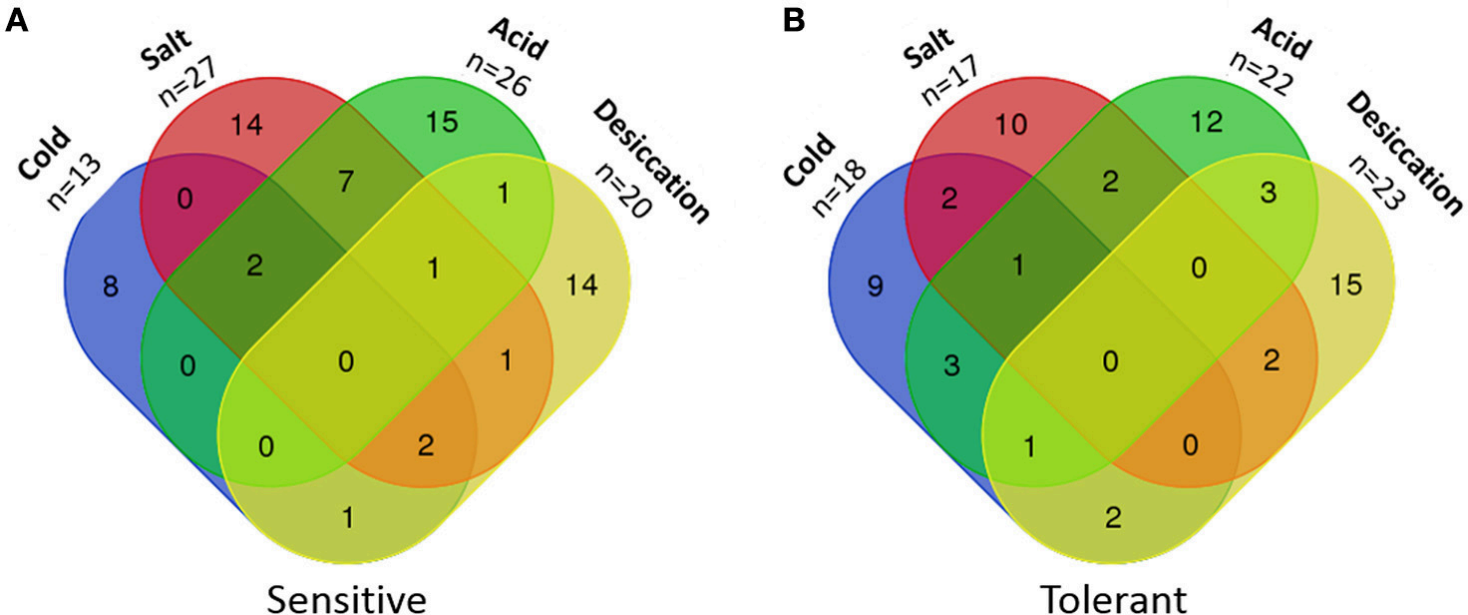
**Numbers of *L. monocytogenes* isolates with multiple sensitivities or tolerances to food-related stresses**. **(A)** Sensitive isolates. **(B)** Tolerant isolates.

Five isolates were classified as sensitive to three out of four stresses and 10 isolates were sensitive to two stresses, seven of which were salt and acid sensitive (Figure 2A). Only two isolates were classified as tolerant to three out of the four stresses and another 14 isolates were tolerant to two of the four stresses (Figure 2B). Twenty additional isolates displayed a total of 16 combinations of overlapping sensitive and tolerant phenotypes (Table S1). The most common overlapping phenotypes were salt and acid sensitive (*n* = 10), salt sensitive and desiccation tolerant (*n* = 6), cold and acid tolerant (*n* = 5), and cold tolerant and salt sensitive (*n* = 5).

Isolates designated sensitive, intermediate or tolerant to one stress were analyzed to determine if they significantly differed in their tolerances to other stresses. When grown in 6% NaCl, acid tolerant isolates had larger std-μ_max_ values compared to acid sensitive isolates (*p* = 0.03, x 1.02 vs. 0.95). Similarly, salt sensitive isolates had smaller std-μ_max_ values (x = 0.80) in BHIB pH 5 compared to intermediate (*p* < 0.0005, x = 1.03) and salt tolerant isolates (*p* = 0.006, x = 1.00).

### Stress tolerances of *L. monocytogenes* lineages, serotypes, and clonal complexes

**Figure 3:**
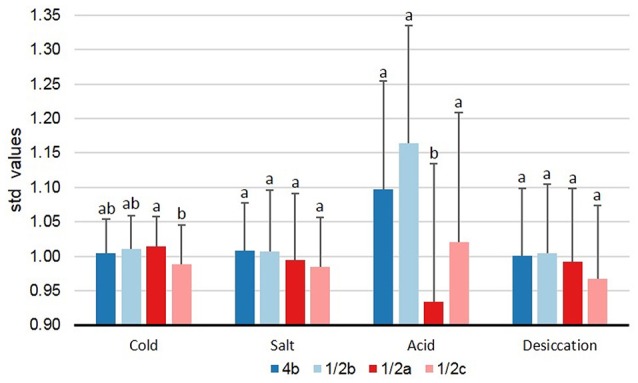
**Levels of tolerance to food-related stresses among *L. monocytogenes* serotypes**. Isolates were evaluated on their ability to survive cold (BHIB at 4°C), salt (BHIB+6% NaCl, 25°C), acid (BHIB pH 5, 25°C), and desiccation stress (33% RH for 3 days at 20°C followed by rehydration with BHIB at 30°C). Error bars represent standard deviations (*n* − 1). Serotypes with different letters within the same stress are significantly different (*p* < 0.05). std-μ_max_, standardized maximum growth rate; std-TRG, standardized time to detectable regrowth; BHIB, brain heart infusion broth.

Between lineages, the only significant difference observed was that LI isolates had significantly larger std-μ_max_ values in BHIB pH 5 than LII isolates (*p* < 0.0005, x = 1.13 vs. x = 0.94). Additional significant differences were observed between serotypes. At 4°C, serotype 1/2a isolates had significantly larger (*p* = 0.017) std-μ_max_ values compared to serotype 1/2c isolates (Figure 3). In support of this, serotype 1/2a isolates accounted for 61% of the cold tolerant isolates and only 31% of cold sensitive isolates compared to a 55% prevalence of this serotype in the collection (Table 1). Similarly, serotype 1/2c isolates accounted for 38% of cold sensitive isolates despite a 15% overall prevalence in the collection (Table 1). When isolates were grown in 6% NaCl, no significant differences were observed between serotypes (Figure 3), however, 71% of salt tolerant and 67% of salt sensitive isolates were serotype 1/2a isolates, relative again to a prevalence of 55% in the collection (Table 1).

In BHIB pH 5, serotype 1/2a isolates had significantly smaller (*p* = 0.027) std-μ_max_ values than serotypes 1/2b, 1/2c, and 4b (Figure 3). In agreement with these findings, 85% of acid sensitive isolates were serotype 1/2a whereas only 32% of acid tolerant isolates were serotype 1/2a (Table 1). No significant differences (*p* > 0.05) were observed between serotypes with respect to desiccation stress std-TRGs (Figure 3).

**Figure 4:**
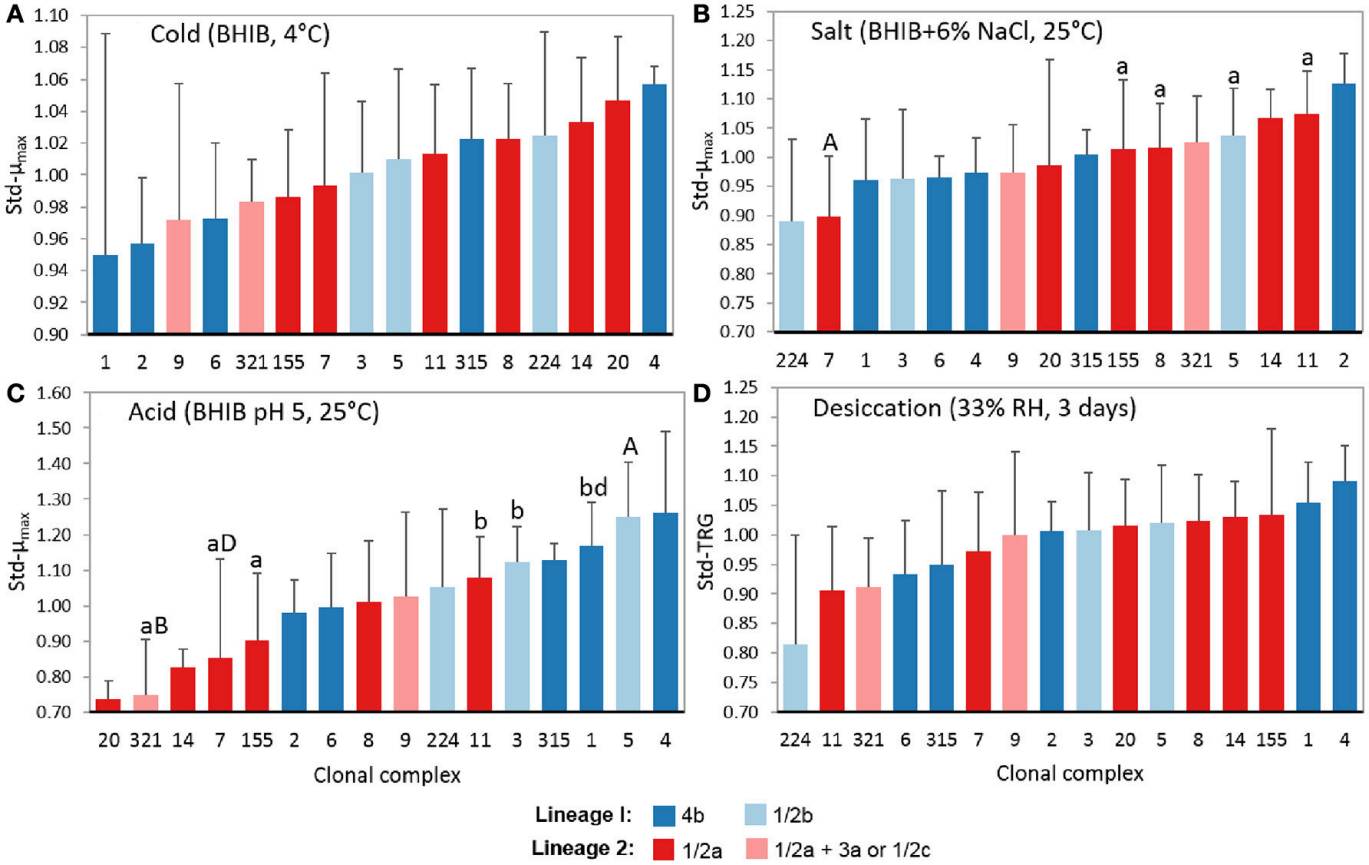
**Levels of tolerance to food-related stresses of different *L. monocytogenes* clonal complexes**. Isolates were evaluated on their ability to survive **(A)** cold (BHIB at 4°C), **(B)** salt (BHIB+6% NaCl, 25°C), **(C)** acid (BHIB pH 5, 25°C), and **(D)** desiccation stress (33% RH for 3 days at 20°C followed by rehydration with BHIB at 30°C). Error bars represent standard deviations (*n* − 1) of standardized model values. CCs with different cases of the same letter are significantly different (*p* < 0.05). std-μ_max_, standardized maximum growth rate; std-TRG, standardized time to detectable regrowth; BHIB, brain heart infusion broth.

Beyond the stress tolerances of lineages and serotypes, some significant differences were also detected between CCs. As a minimum of six isolates per CC were needed to ensure a power >0.80 for ANOVA results, statistical analyses were only performed using CCs 1, 3, 5, 6, 7, 8, 9, 11, 155, and 321. Figure 4 shows the average levels of cold (std-μ_max_), salt (std-μ_max_), acid (std-μ_max_), and desiccation (std-TRG) tolerance among CCs with three or more isolates. At At 4°C, no significant differences were found between the growth rates of different CCs, however, it was interestingly to see that CCs associated with 4b isolates had both the lowest and highest average average std-μ_max_ values at 4°C, demonstrating why stress tolerance differences were not observed between this serotype and others at 4°C (Figure 4A).

In 6% NaCl, CC7 (1/2a) isolates had significantly (*p* < 0.05) smaller std-μ_max_ values compared to CCs 5 (1/2b), 8 (1/2a), 11 (1/2a), and 155 (1/2a; Figure 4B). This highlights the range of salt tolerances between CCs within the same serotype and again explains why no significant differences were observed at the serotype level for salt tolerance. In support of the results shown in Figure 4B, 67% of CC2 isolates were salt tolerant while 50% of CC224, 47% of CC7, and 22% of CC9 isolates were salt sensitive (Table 2).

In BHIB pH 5, CC5 (1/2b) isolates exhibited significantly (*p* < 0.05) larger std-μ_max_ values than CCs 7, 155, and 321, and CC321 isolates additionally had smaller (*p* < 0.05) std-μ_max_ values compared to CCs 1, 3, and 11 (Figure 4C). CC1 (4b) isolates also had significantly (*p* = 0.02) smaller std-μ_max_ values compared to CC7 (1/2a) isolates. From Figure 4C it can be seen that lineage I isolates were more acid tolerant than LII isolates, as the five CCs with the highest average std-μ_max_ values in BHIB pH 5 were all from LI while the five CCs with the lowest average std-μ_max_ values belonged to LII (predominantly 1/2a isolates). Notably, 57% of CC5 and 67% of CC4 isolates were acid tolerant while 83% of CC321, 67% of CC20, and 29% of CC7 isolates were acid sensitive (Table 2).

No significant differences were found between the desiccation stress std-TRGs of different CCs (Figure 4D). Nevertheless, CC224 (1/2b) had the smallest average std-TRGs and correspondingly, 75% of these isolates were classified as desiccation tolerant. CC11 (1/2a) had the next smallest std-TRGs while CCs 1 and 4 (both 4b) had the two largest average std-TRGs.

### Associations between plasmid harborage and stress tolerances

**Figure 5:**
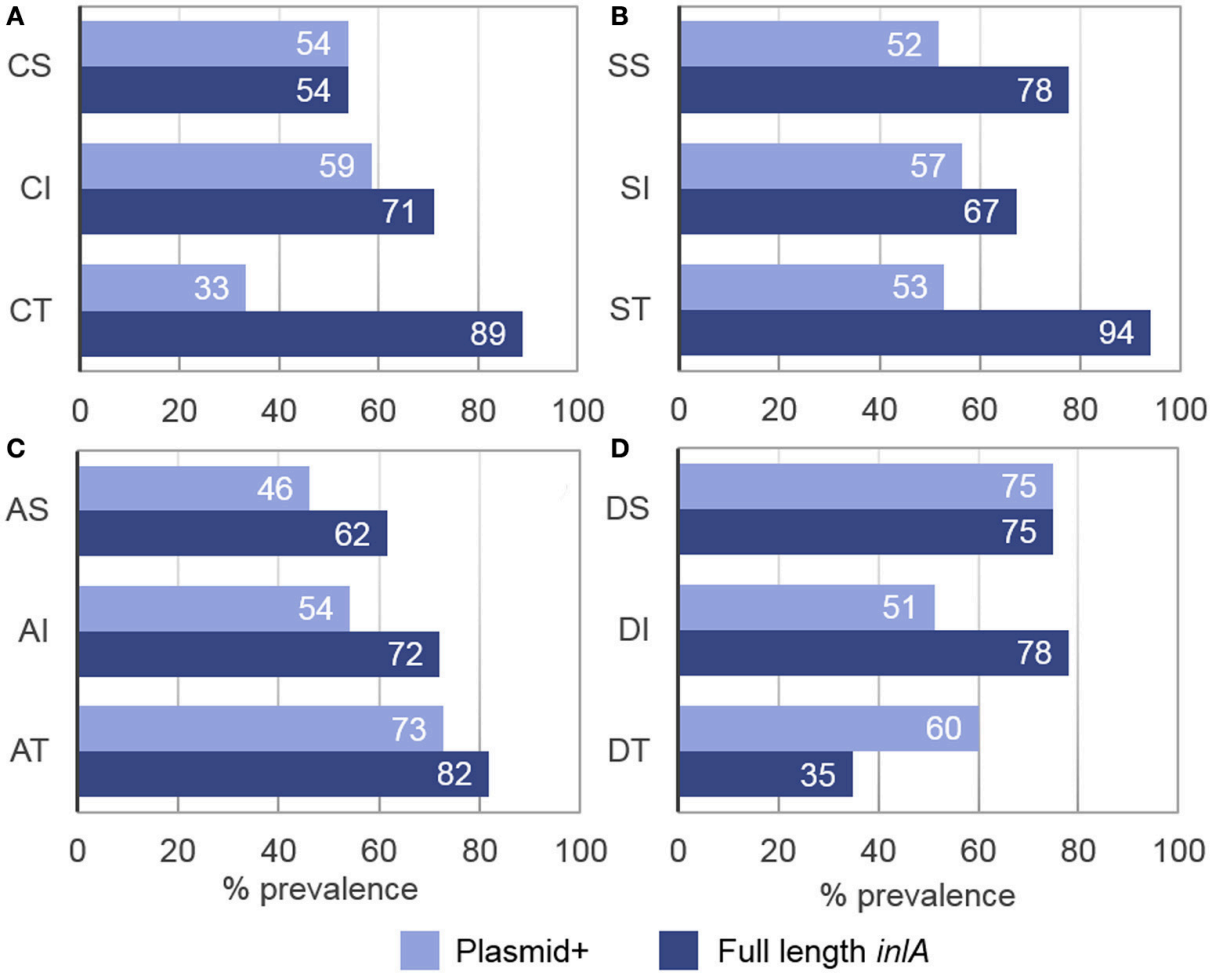
**Prevalence of full length *inlA* and plasmid harborage among *L. monocytogenes* stress tolerance phenotypes. (A)** Cold sensitive (CS), intermediate (CI), and tolerant (CT) isolates. **(B)** Salt sensitive (SS), intermediate (SI), and tolerant (ST) isolates. **(C)** Acid sensitive (AS), intermediate (AI), and tolerant isolates (AT). **(D)** Desiccation sensitive (DS), intermediate (DI), and tolerant (DT) isolates. Full length *inlA* and the presence of a plasmid were observed in 72 and 55% of all isolates, respectively.

Although plasmids were identified in 55% of all isolates, a higher percentage of plasmid carriers were observed among acid tolerant (73%), desiccation sensitive (75%), and desiccation tolerant (60%) isolates as compared to cold tolerant (33%) and acid sensitive (46%) isolates (Figure 5). Within LII, plasmid-positive isolates had smaller std-μ_max_ values at 4°C (*p* = 0.024, x = 1.00 vs. 1.02) and larger std-μ_max_ (*p* < 0.0005, x = 1.01 vs. 0.86) values when grown in BHIB pH 5 compared to their plasmid-free counterparts. No significant differences were found between the stress tolerance levels of LI plasmid-harboring and plasmid-free isolates. It was, however, observed that isolates containing *repA* group 1 plasmids; which were significantly (*p* < 0.0005) smaller than group 2 plasmids, had smaller std-μ_max_ (*p* = 0.002, x = 0.98 vs. 1.04) values in 6% NaCl.

### Associations between *inlA* profiles and stress tolerances

Full length *inlA* was observed in 72% of isolates, where a higher percentage of the intact gene prevailed among cold (89%), salt (94%), and acid (82%) tolerant isolates while a lower prevalence was detected among desiccation tolerant isolates (35%; Figure 5). Statistically, isolates with full length *inlA* had significantly larger std-μ_max_ values at 4°C than isolates with an *inlA* PMSC (*p* = 0.001, x = 1.01 vs. 0.97). Additionally, serotype 4b isolates possessing a three-codon deletion in *inlA*, had significantly shorter desiccation stress std-TRGs (*p* = 0.002 x = 0.94 vs. 1.05) compared to serotype 4b isolates with full length *inlA*. No significant associations were found between *inlA* profiles and salt or acid stress tolerance.

### Associations between stress tolerances and the presence of SSI1 or LGI1

In 6% NaCl, isolates containing SSI-1 had significantly smaller std-μ_max_ values (*p* = 0.004, x = 0.98 vs. 1.03) than isolates without SSI-1 though this difference was not large. To determine potential associations between LGI1 harborage and stress tolerance, serotype 1/2a isolates containing the island were compared to other 1/2a LGI1-negative isolates but similar to SSI-1, no significant differences in stress tolerances were detected.

### SNV analyses of stress-sensitive and tolerant isolates

**Figure 6:**
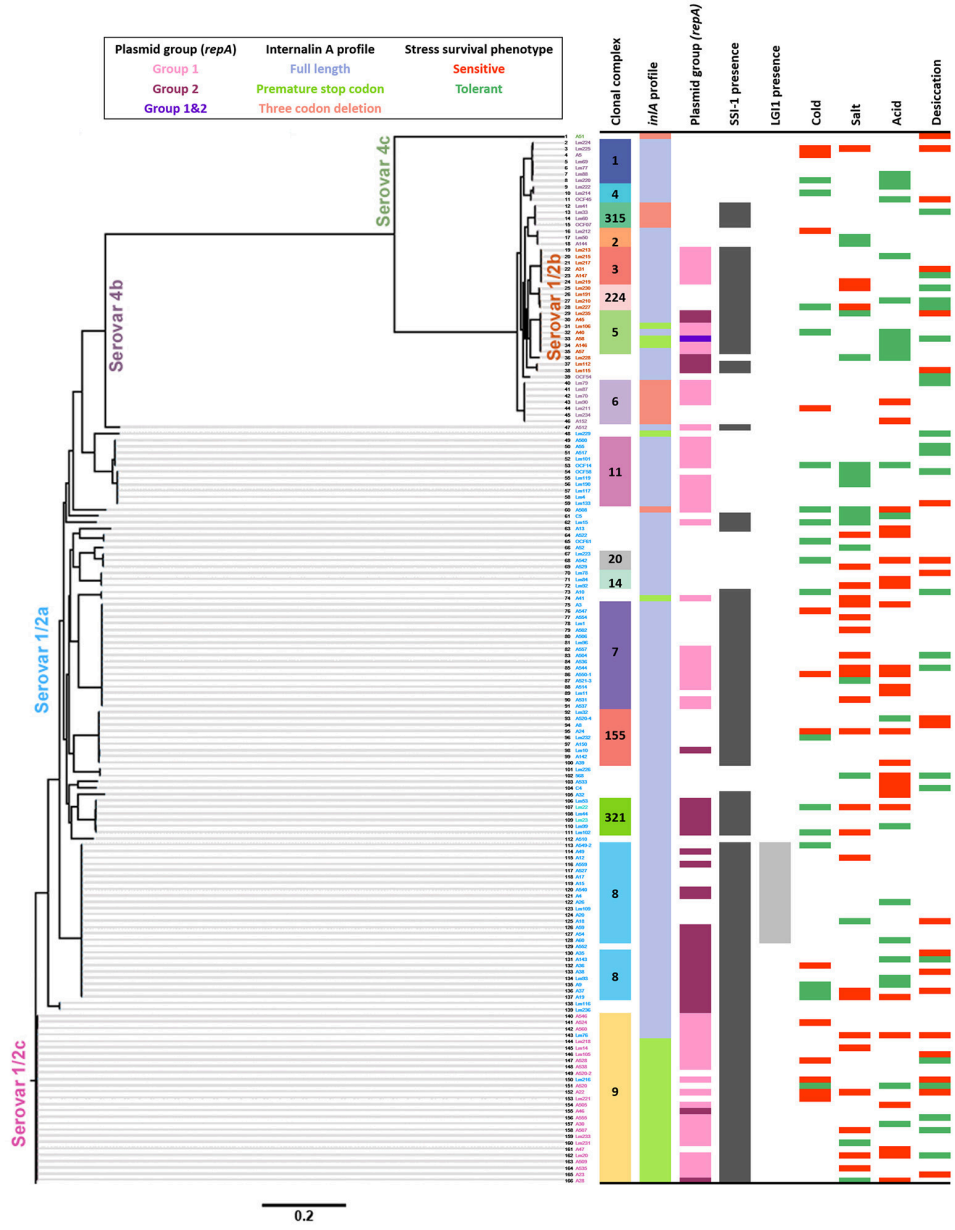
**Whole genome single nucleotide variant (SNV) phylogeny of 166 *L. monocytogenes* isolates and their associated genetic characteristics and stress tolerance phenotypes**. The scale at the bottom indicates the substitutions per SNV.

Figure 6 shows a whole genome SNV phylogeny of all 166 *L. monocytogenes* isolates with their corresponding genetic and phenotypic properties. In this figure, groups of closely related isolates that share the same phenotypes can be seen. However, also shown are several cases where neighboring isolates have opposing stress tolerances. Of particular interest was whether specific SNVs could be related to isolates possessing the same stress tolerance phenotypes, however, none were detected to be uniquely shared among stress tolerant isolates that weren't also seen in intermediate or sensitive isolates. Among stress sensitive isolates, unique SNVs shared by subsets of isolates were identified, but no single SNV was prevalent among >4 isolates from the same stress sensitive phenotype group. In contrast, a large number of SNVs were uniquely observed for one or two isolates from the same stress sensitive group, causing frameshifts, premature stop codons, loss of start codons, or missense variants. Information regarding the SNVs identified among all sensitive and tolerant isolates are presented in Tables S2–S9. Notably, a number of stress sensitive isolates contained different PMSCs in several σ^B^ regulator genes. A cold and desiccation sensitive isolate contained a PMSC in *rsbS* as did two other desiccation sensitive isolates. Furthermore, an additional cold sensitive isolate contained a PMSC in *rsbV*, and two desiccation sensitive isolates contained PMSCs in *rsbU*.

### Genomic islands of stress-sensitive and tolerant isolates

All *L. monocytogenes* isolates were predicted to harbor 1,318 genomic islands in total, resulting in an average of eight genomic islands per genome. These islands were clustered into 200 groups of similar sequences. The conservation of genomic island groups across *L. monocytogenes* lineages ranged from unique to a single isolate to conserved in 97 isolates (58%). Most frequently, intermediate groups conserved in subsets of monophyletic isolates were observed, as can be expected from a combination of vertical inheritance of the genomic islands with further modifications by mutation, insertions, and deletions. The clustering of *L. monocytogenes* isolates based on the presence or absence of groups of genomic islands reflected their phylogenetic proximity and did not relate particular genomic island content in isolates with the exhibition of similar phenotypes. Indeed, no single genomic island was found to occur in a large proportion of strains with a given phenotype (Figure S1).

## Discussion

### *L. monocytogenes*' tolerance to food-related stresses differs between and within lineages, serotypes, and clonal complexes

#### Cold stress

*L. monocytogenes*' ability to grow at refrigeration temperatures highlights this pathogen as a concern for the food industry and consumers alike. However, it is known that *L. monocytogenes* strains can largely differ in their ability to adapt to cold stress. In the present study, it was found that serotypes 1/2a and 1/2b were on average more cold-tolerant than serotypes 4b and 1/2c. Other cold growth studies have also reported serotype 1/2a strains to be more cold tolerant than serotype 4b strains (Junttila et al., 1988; Buncic et al., 2001; Lianou et al., 2006) though similar to the current findings, many of these differences were not statistically significant due to strain to strain variations. Barbosa et al. (1994) reported that out of 39 *L. monocytogenes* strains, Scott A, a 4b clinical isolate, grew the slowest at 4°C and that 1/2a strains grew the fastest followed by 1/2b, and 4b. Similarly, in De Jesús and Whiting (2003), LII isolates (all serotype 1/2a) exhibited the shortest LPDs at 5°C followed by LI and then LIII isolates. Researchers have suggested that LII strains may be able to survive better under food-related stresses due to an enhanced ability to acquire advantageous mutations and extrachromosomal DNA compared to LI strains which typically have more conserved genomes (Orsi et al., 2007, 2008, 2011; Ragon et al., 2008; Dunn et al., 2009). Certain stress response genes, predominantly involved in membrane transport and cell wall structure (Doumith et al., 2004), have also been reported to be present in LII isolates but absent among LI isolates (Borucki and Call, 2003; Call et al., 2003; Zhang et al., 2003; Doumith et al., 2004; Chan and Wiedmann, 2008). Given the critical roles of these structures in allowing bacteria to adapt and tolerate numerous stresses (Annous et al., 1997; Verheul et al., 1997; Klein et al., 1999; Weber et al., 2001; Álvarez-Ordóñez et al., 2008), it is not surprising that *L. monocytogenes* lineages and serotypes can behave differently under certain stresses. The alternative sigma factor, σ^C^, and *lmo1078*, encoding a UDP-glucose pyrophosphorylase, are examples of genes with reported roles in *L. monocytogenes* cold tolerance, that are present in LII strains but absent in LI and serotype 4b strains, respectively (Chassaing and Auvray, 2007; Chan and Wiedmann, 2008). These absences may partly explain the overall reduced cold tolerance of serotype 4b isolates respective to 1/2a isolates. However, they do not appear to be necessary for adequate cold growth as in the present study some 4b isolates were also classified as cold tolerant.

#### Salt stress

LI strains have been shown to be more salt tolerant than LII strains (Bergholz et al., 2010) and serotype 4b strains to be more salt tolerant than serotype 1/2a and 1/2b strains (Van Der Veen et al., 2008; Bergholz et al., 2010; Ribeiro et al., 2014). In the present study, no significant differences were found between the growth rates of different serotypes in 6% NaCl, however, five times as many 4b isolates were classified as salt tolerant as were classified as salt sensitive. Additionally, despite a 55% prevalence of serotype 1/2a isolates in our collection, isolates of this serotype accounted for 71% of salt tolerant isolates but also for 67% of salt sensitive isolates, indicating a broad range of salt tolerance among isolates from this serotype. These differences were found to be associated with specific 1/2a CCs, notably, CC7 isolates were on average the most salt sensitive CC and significantly differed from the 1/2a CCs 8 and 11 with most 1/2a salt tolerant isolates belonging to CC11.

#### Acid stress

LI isolates tolerated acid stress conditions significantly better than LII isolates. Specifically, serotype 1/2a showed higher sensitivity to acidity. This trend was also clear when the acid tolerance of individual CCs was investigated. Van Der Veen et al. (2008), also reported that 4b (LI) isolates had enhanced acid tolerance relative to 1/2a isolates. The authors hypothesized that the increased survival of 4b strains may in part be due to the presence of *ORF2110* which encodes a putative serine protease similar to HtrA. This protein has been shown to be important for growth at low pH, high osmolarity, and high temperatures (Wonderling et al., 2004; Stack et al., 2005; Wilson et al., 2006). Though this gene may contribute to the overall acid tolerance of 4b isolates, some acid sensitive 4b isolates were also identified. Similarly, despite serotype 1/2a isolates having relatively low acid tolerance overall, some 1/2a isolates were also acid tolerant, highlighting the importance of not overgeneralizing isolate phenotypes based on the trends seen for their sero- or sequence type.

#### Desiccation stress

Desiccation tolerance reflects a bacteria's ability to survive on a surface for extended periods of time with little access to nutrients and water. As so, desiccation tolerance is believed to be associated with *L. monocytogenes*' ability to persist in food production plants (Vogel et al., 2010) and subsequently cross-contaminate food products. To date, surprisingly little research has been conducted regarding *L. monocytogenes*' desiccation survival and that which does exist has focused primarily on factors influencing the survival of a small number of isolates (Vogel et al., 2010; Hansen and Vogel, 2011; Takahashi et al., 2011; Hingston et al., 2013; Overney et al., 2016; Zoz et al., 2016). The current study is the first to our knowledge, to compare the desiccation tolerance of *L. monocytogenes* isolates from multiple serotypes. No significant differences were found between serotypes or CCs however, some prominent trends were observed. Serotypes 1/2c and 1/2b were on average the most desiccation tolerant, followed by 4b and 1/2a. More specifically, CC224 (1/2b) isolates had the highest levels of desiccation survival. Interestingly, a large listeriosis outbreak in Denmark, which resulted in 41 illnesses and 17 deaths, was linked to the consumption of deli meat contaminated with a CC224 strain (Kvistholm Jensen et al., 2016). Though there is not enough evidence to suggest that all CC224 strains are desiccation tolerant, it is possible that long-term desiccation survival may have contributed to the occurrence of this outbreak. Another interesting finding from the present study was that the most desiccation sensitive isolate, deviating more than 4.5 *SD* from the median, was a CC193 (serotype 1/2a) isolate. Since this was the only CC193 isolate in our collection, it would be interesting to analyze additional isolates from this CC to determine if this sequence type is associated with a high degree of desiccation sensitivity.

### Certain genetic elements are associated with the stress tolerance of *L. monocytogenes*

#### Plasmids

The presumptive presence of a plasmid(s) was detected in 55% of our isolates which is comparable to other studies where rates of plasmid isolation have ranged from 0 to 79% with an overall average of around 30% (Perez-Diaz et al., 1982; Kolstad et al., 1992; Lebrun et al., 1992; Peterkin et al., 1992; McLauchlin et al., 1997). In agreement with earlier work, we also observed that plasmid DNA was more prevalent among LII isolates than LI isolates (Kolstad et al., 1992; Lebrun et al., 1992; McLauchlin et al., 1997; Margolles and de los Reyes-Gavilán, 1998; Orsi et al., 2011).

Kuenne et al. (2010) discovered that *L. monocytogenes* plasmids could be divided into two phylogenetic groups based on their *repA* sequences and that group 2 plasmids (77–83 kb) were larger than those belonging to group 1 (32–57 kb). Here plasmid *repA* sequences also formed two distinct phylogenetic groups and in agreement with Kuenne et al. (2010), group 2 plasmids were significantly larger (55–100 kb) than group 1 plasmids (26–88 kb). Notably, one serotype 1/2b isolate contained two plasmids of similar sizes (62 and 69 kb) but belonging to different *repA* groups. Though rare, the presence of two plasmids has been reported in other *Listeria* spp. isolates (Earnshaw and Lawrence, 1998; Margolles and de los Reyes-Gavilán, 1998).

Our results showed that among LII isolates, which exhibited higher rates of plasmid harborage than LI isolates, plasmid harborage was associated with significantly enhanced acid tolerance but also cold sensitivity. Studies have shown that plasmid harborage and subsequent replication increases the metabolic demands of cells, leading to decreased growth rate relative to plasmid-free strains (reviewed in Diaz Ricci and Hernández, 2000). However, depending on the genes contained on a plasmid, plasmid-harborage can also provide cells with a growth advantage when exposed to certain conditions. In this study, isolates with the larger *repA* group 2 plasmids were significantly more salt-tolerant than isolates that harbored the smaller *repA* group 1 plasmids, collectively suggesting that plasmid harborage may be a hindrance to *L. monocytogenes* during replication at low temperatures but provide an advantage when exposed to acid and salt stress conditions. Furthermore, the observation that isolates containing larger plasmids had higher levels of salt tolerance suggests that these plasmids may contain additional genes that are beneficial for adaptation to high osmolarity environments.

To date, plasmids acquired by *L. monocytogenes* have been shown to contain genes that confer resistance to benzalkonium chloride (*bcrABC*; Elhanafi et al., 2010; Rakic-Martinez et al., 2011; Katharios-Lanwermeyer et al., 2012), cadmium (*cadA2, cadAC*; Lebrun et al., 1992; Rakic-Martinez et al., 2011; Katharios-Lanwermeyer et al., 2012) and antibiotics including chloramphenicol, clindamycin, erythromycin, streptomycin, and tetracycline (Poyart-Salmeron et al., 1990; Hadorn et al., 1993). *Listeria* spp. plasmids also commonly contain several other uncharacterized efflux pumps (MDR, SMR, MATE; Gibson and Parales, 2000; Masaoka et al., 2000; Boylan et al., 2006; Kuroda and Tsuchiya, 2009), as well as oxidative stress response genes (peroxidases, reductases; Kuenne et al., 2010) but their exact roles in stress tolerance have yet to be investigated. In other bacterial species, multidrug efflux pumps have been linked to stress response, virulence, and quorum sensing (reviewed in Li and Nikaido, 2009). Ma et al. (1995) reported that transcription of a MDR pump (*acrAB*) in *E. coli* increased in response to fatty acids, ethanol, high salt, and cellular transitioning into stationary phase. Among the putative plasmid associated contigs a wide variety of different genes were identified including those encoding cell surface proteins, lipoproteins, secretion pathways, heavy metal transporters, transcription regulators, general stress proteins (CplB, ClpL), NADH oxidoreductases, a glycine betaine transport permease (ProW), and the multidrug resistance proteins EbrA and EbrB among others. All group 2 plasmids shared a general secretion pathway protein and a cell surface protein. Other genes identified among many but not all group 2 plasmids included those which encoded DNA topoisomerase III, a membrane-bound protease, a NLP/P60 family lipoprotein, an NADH peroxidase, and a type IV secretory pathway. Further investigations are currently focusing on whether these genes or others with no known function contribute to *L. monocytogenes*' acid and salt tolerance.

Lastly, it should be highlighted that plasmids with 99–100% nucleotide identity were found in isolates from different serotypes, provinces (Alberta and British Columbia), and countries (Canada and Switzerland). One plasmid was observed in 26 isolates, which strongly suggests that *L. monocytogenes'* benefits from its presence. The occurrence of the same plasmid in multiple food-related isolates from different regions also suggests that bacteria are frequently transported between places of food production, possibly alongside imported raw materials. On the contrary, it is particularly interesting that other plasmids were conserved among specific CCs, serotypes, provinces, and countries.

#### Full length *inlA*

Full length *inlA* profiles were observed among 92% of serotype 1/2a isolates, 83% of serotype 1/2b isolates, and 12% of serotype 1/2c isolates, reflecting what has been previously observed (Jonquieres et al., 1998; Jacquet et al., 2004; Rousseaux et al., 2004; Nightingale et al., 2005; Felicio et al., 2007; Orsi et al., 2007; Ragon et al., 2008). Additionally, 44% of 4b isolates contained a 3-codon deletion that unlike *inlA* PMSCs, is not associated with attenuated virulence (Kovacevic et al., 2013; Kanki et al., 2015).

The present study results showed that full length *inlA* profiles were more prevalent among cold, salt, and acid tolerant isolates compared to their sensitive counterparts. Also, isolates with full length *inlA* profiles were significantly more cold tolerant than isolates containing *inlA* PMSCs. Kovacevic et al. (2013) were the first to report that cold tolerant isolates more likely possess full length *inlA* than intermediate and cold sensitive isolates. This increased stress tolerance has now been shown to extend to salt and acid tolerance, making it reasonable to hypothesize that full length *inlA* may participate in *L. monocytogenes*' stress response. When bacteria are exposed to unfavorable conditions, their cell envelope is the first line of defense. It is possible that the absence of cell wall anchored InlA proteins may alter cell-surface characteristics, leaving cells more susceptible to certain environmental stresses. Interestingly, only a small percentage of desiccation tolerant isolates contained full length *inlA* while serotype 4b isolates with full length *inlA* profiles had significantly impaired desiccation survival relative to those with a 3-codon deletion. Again, it is suspected that the structure of *inlA* may influence *L. monocytogenes*' desiccation tolerance, this time with the full length form possibly imparting a disadvantage. Other researchers have also detected associations between internalin mutations and certain phenotypes. In Hingston et al. (2015), *inlC* was identified as the interrupted gene in a desiccation tolerant transposon mutant and Franciosa et al. (2009) found that strains possessing a truncated *inlA* protein formed increased levels of biofilm. Similarly, transposon mutants containing an interrupted internalin A, B, or H gene, formed thicker biofilms relative to the wildtype (Piercey et al., 2016). Together, these findings along with those presented in this study, emphasize a need for more research regarding the potential roles of internalins in other processes other than virulence.

#### SSI-1

Stress survival islet 1 (SSI-1) is a five-gene cluster which has previously been shown via mutagenesis studies to enhance *L. monocytogenes* tolerance to acid, salt, and low temperature conditions (Cotter et al., 2005; Ryan et al., 2010). On the other hand, Arguedas-Villa et al. (2014) found no significant differences in cold tolerance between naturally occurring *L. monocytogenes* isolates with and without SSI-1. In the present study, it was found that SSI-1-positive isolates showed no enhanced cold, acid, salt, and desiccation stress tolerances relative to SSI-1 negative isolates. It is possible that any positive influence of SSI-1 on the stress tolerance of *L. monocytogenes*' may be masked by the presence of other genetic elements when comparing large collections of isolates as opposed to a mutant and its wildtype strain.

#### LGI1

LGI1 is a *Listeria* 50 kb genomic island that was first identified in Canadian CC8 isolates associated with a large 2008 listeriosis outbreak involving contaminated deli meats and resulting in 22 fatalities (Gilmour et al., 2010). Since then, LGI1 has been identified in other CC8 *L. monocytogenes* isolates from Canada (Kovacevic et al., 2013) but not from other countries (Althaus et al., 2014). In agreement with these studies, the presence of LGI1 was only detected in Canadian isolates from CC8. However, instead of all LGI1+ isolates being ST120 as previously reported, two novel CC8 STs (ST1022 and 1025) were also associated with LGI1 harborage. The conservation of LGI1 among Canadian isolates and its association with a fatal outbreak has led to heightened interest in the putative functions of the genes located on this island including those encoding putative type II and type IV secretion systems, pilus-like surface structures, a multidrug efflux pump homolog (EmrE), and an alternative sigma factor (Gilmour et al., 2010). Recently, Kovacevic et al. (2015) reported that deletion of LGI1 genes with putative efflux (*emrE*), regulatory (*lmo1852*), and adhesion (*sel1*) functions, had no impact on the tolerance of *L. monocytogenes*' to acid, cold, or salt, but that deletion of *emrE* increased susceptibility to quaternary ammonium-based sanitizers. Based on these findings, it was investigated whether the presence or absence of the whole LGI1 island could be associated with stress tolerance differences between CC8 isolates however, no significant differences were identified. Consequently, LGI1 had no major influence on *L. monocytogenes*' ability to adapt to the food-related stresses evaluated in the present study. However, it is possible that the island contributes in other ways to the persistence of CC8 Canadian isolates in food processing environments in addition to the role of *emrE* in sanitizer resistance.

### SNVs associated with stress tolerance phenotypes

An important finding from this study was that closely related isolates from within the same clonal complexes exhibited opposing stress tolerances, suggesting that minor genetic differences can also exert great impact on stress tolerance phenotypes. This was observed in a study by Hoffmann et al. (2013), where a single thymine deletion in the σ^A^-like promoter region of *betL*, encoding an osmolyte transporter specific for betaine uptake, dramatically increased *betL* transcription, and hence the osmo- and chill-tolerance. Karatzas et al. (2003) reported that a spontaneous high hydrostatic pressure tolerant *L. monocytogenes* mutant of Scott A, contained a single codon deletion in *ctsR*, a negative regulator of several heat shock and general stress proteins, that also conferred increased thermo-tolerance and resistance to H_2_O_2_. Additionally, in Metselaar et al. (2015) a number of spontaneous acid tolerant mutants were found to contain SNVs in the ribosomal protein gene *rpsU*. None of these aforementioned mutations were detected in the present study.

Analyses to determine if unique SNVs could be detected among isolates from individual stress tolerance phenotype groups were also performed. Studies identifying the genetic basis of phenotypic traits using the variation within natural populations are known as a genome-wide association studies (GWAS). While GWAS have been effective for identifying mutations responsible for phenotypic traits in humans, the clonal nature of bacterial replication where mutations can reach a high frequency on a single genetic background, makes it difficult to distinguish mutations responsible for an observed phenotype (Read and Massey, 2014; Falush, 2016). As a result, bacterial molecular epidemiology has focused on identifying clonal lineages with particular phenotypic properties rather than identifying the specific genetic variants responsible. Recently, Earle et al. (2016) used a GWAS approach to successfully identify genes and genetic variants underlying resistance to 17 antimicrobials in over 3000 isolates of taxonomically diverse clonal and recombining bacteria. While these results show the potential of bacterial GWAS, antimicrobial resistance is usually gained during antimicrobial exposure and thus it is more likely that the traits evolve on multiple independent backgrounds making them easier to detect (Falush, 2016). To date, the identification of mutations responsible for more complex phenotypes such as those evaluated in the present study, remain challenging.

Our analyses did not detect any unique SNVs among more than one isolate from the same stress tolerant group, suggesting homoplasy among stress tolerant phenotypes where mutations evolve independently to confer tolerance. On the contrary, a few different SNVs were identified among four or fewer isolates from the same stress sensitive groups. Notably, the cold sensitive phenotypes of two isolates may be associated with PMSCs detected in the σ^B^ regulator genes *rsbS* and *rsbV* (Voelker et al., 1995). In support of this hypothesis, deletion of *rsbV* in a *L. monocytogenes* mutagenesis study resulted in impaired cold stress tolerance (Chan et al., 2008). Three desiccation sensitive isolates also contained different PMSCs in *rsbS*, including one isolate which was also cold sensitive. An additional two desiccation sensitive isolates contained different PMSCs in another σ^B^ posttranscriptional regulator, *rsbU*. Given the importance of σ^B^ in *L. monocytogenes*' adaptation to several environmental stresses (Wiedmann et al., 1998; Kazmierczak et al., 2003), it is possible that these mutations contributed to the reduced desiccation tolerance of these isolates. In fact, in Huang et al. (2015) an *L. monocytogenes sigB* mutant demonstrated reduced desiccation survival relative to the wildtype strain.

### Genomic islands

In this study, specific genomic islands could not be exclusively associated with a particular stress tolerance phenotype. This is to be expected as genomic islands often contain virulence factors, and in general these are overrepresented in genomic islands as compared to the chromosome (Sui et al., 2009). In *L. monocytogenes* in particular, genomic islands have been associated with virulence, heavy metal resistance, and benzalkonium chloride efflux (Gilmour et al., 2010; Kuenne et al., 2010; Kovacevic et al., 2015). SSI-1 as described above, has been previously associated with *L. monocytogenes*' stress response, but was not significantly correlated with the stress tolerance phenotypes examined in the present study.

## Conclusions

In summary, *L. monocytogenes*' tolerances to certain food-related stresses differs between serotypes as well as CCs with the latter being a better predictor of isolate salt and acid tolerance but not of cold and desiccation tolerance. To the best of our knowledge, this is the first study to evaluate the stress tolerance of different *L. monocytogenes* CCs. Other noteworthy findings include potential relationships between the presence of full length *inlA* and enhanced cold tolerance and the presence of a plasmid and enhanced acid tolerance. On the contrary, the presence of genomic islands including SSI-1 and LGI1, provided isolates with no noticeable advantages under the stresses evaluated in this study. Additional research is needed to confirm the potential roles of full length *inlA* and plasmid associated genes in *L. monocytogenes*' response to various stresses.

A whole genome SNV phylogeny of isolate assemblies identified a number of unique SNVs shared by up to four stress sensitive isolates while no common SNVs were observed among stress tolerant isolates. More specifically, six isolates with sensitivity to cold and/or desiccation stress contained PMSCs in σ^B^ regulator genes (*rsbS, rsbU, rsbV*) that may be contributing to these phenotypes.

A number of novel genetic elements were also elucidated in this study including nine new *L. monocytogenes* STs, a new *inlA* PMSC, the absence of a cold stress associated gene (*lmo1078*) in 4b isolates, and several connections between *L. monocytogenes* CCs and the presence/absence or variations of specific genetic elements. For example, SSI-1 was detected in 100% of isolates from specific CCs, certain plasmid groups and sizes were conserved among isolates from the same CCs, and plasmids with 100% identity were found in isolates belonging to the same CCs but from very different geological areas. While our isolate collection represented of a number of *L. monocytogenes* CCs, some of which have been previously identified as common among food-related isolates, other CCs were less prevalent or absent from our study of Canadian and Swiss isolates. This highlights the regional prevalence of certain *L. monocytogenes* genotypes and emphasizes the need for more international collaborative studies.

Collectively, the results suggest that using whole genome sequencing to (1) determine the STs of *L. monocytogenes* food-related isolates and to (2) screen for the presence of genetic elements such as full length *inlA* and a plasmid(s), could help food processors and food agency investigators to quickly identify if isolates are likely to possess enhanced tolerances to certain stresses that may be facilitating their long-term survival/persistence in a food processing environment. The US FDA and CDC are rapidly making whole genome sequencing of foodborne bacterial pathogens a routine part of screening to help link illnesses to contaminated foods and to identify outbreaks earlier. While no one SNV was identified among isolates with the same stress tolerant phenotype, increased sequencing of *L. monocytogenes* isolates in combination with stress tolerance profiling, will enhance the ability to identify genetic elements associated with more stress tolerant strains.

## Author contributions

PH composed the paper and conducted all experiments and statistics. CL assembled and annotated the whole genome sequences. PH, JC, BD, and CB conducted the bioinformatics analyses. JC, VG, FB, TT, KA, LT, and SW provided guidance on study design, statistics, and assisted in writing the paper.

## Funding

This work was funded by an investment agreement between Alberta Innovates—Bio Solutions and the University of British Columbia (FSC-12-030). PH was funded by an NSERC CGS D Scholarship. CB is supported by a fellowship from the Swiss National Science Foundation (P300-PA_164673) and a grant from the Société Académique Vaudoise.

### Conflict of interest statement

The authors declare that the research was conducted in the absence of any commercial or financial relationships that could be construed as a potential conflict of interest.
