# Supplementary material for: Genotypes Associated with Listeria monocytogenes Isolates Displaying Impaired or Enhanced Tolerances to Cold, Salt, Acid, or Desiccation Stress
Source: Front Microbiol. 2017 Mar 8;8:369. doi: 10.3389/fmicb.2017.00369 (PMC5340757; doi:10.3389/fmicb.2017.00369)
Supplement: Supplementary file 10 [file DataSheet1.DOCX]

***Supplementary Material***

**Genotypes Associated with *Listeria monocytogenes* Isolates Displaying Impaired or Enhanced Tolerances to Cold, Salt, Acid, or Desiccation stress**

**Patricia Hingston, Jessica Chen, Bhavjinder Kaur Dhillon, Chad Laing, Claire Bertelli, Victor Gannon, Taurai Tasara, Kevin Allen, Fiona S. L. Brinkman, Lisbeth Truelstrup Hansen, and Siyun Wang^*^**

*** Correspondence:** Siyun Wang [siyun.wang@ubc.ca](mailto:siyun.wang@ubc.ca)

1. **Supplementary Figures and Tables**
   1. **Supplementary Figures**


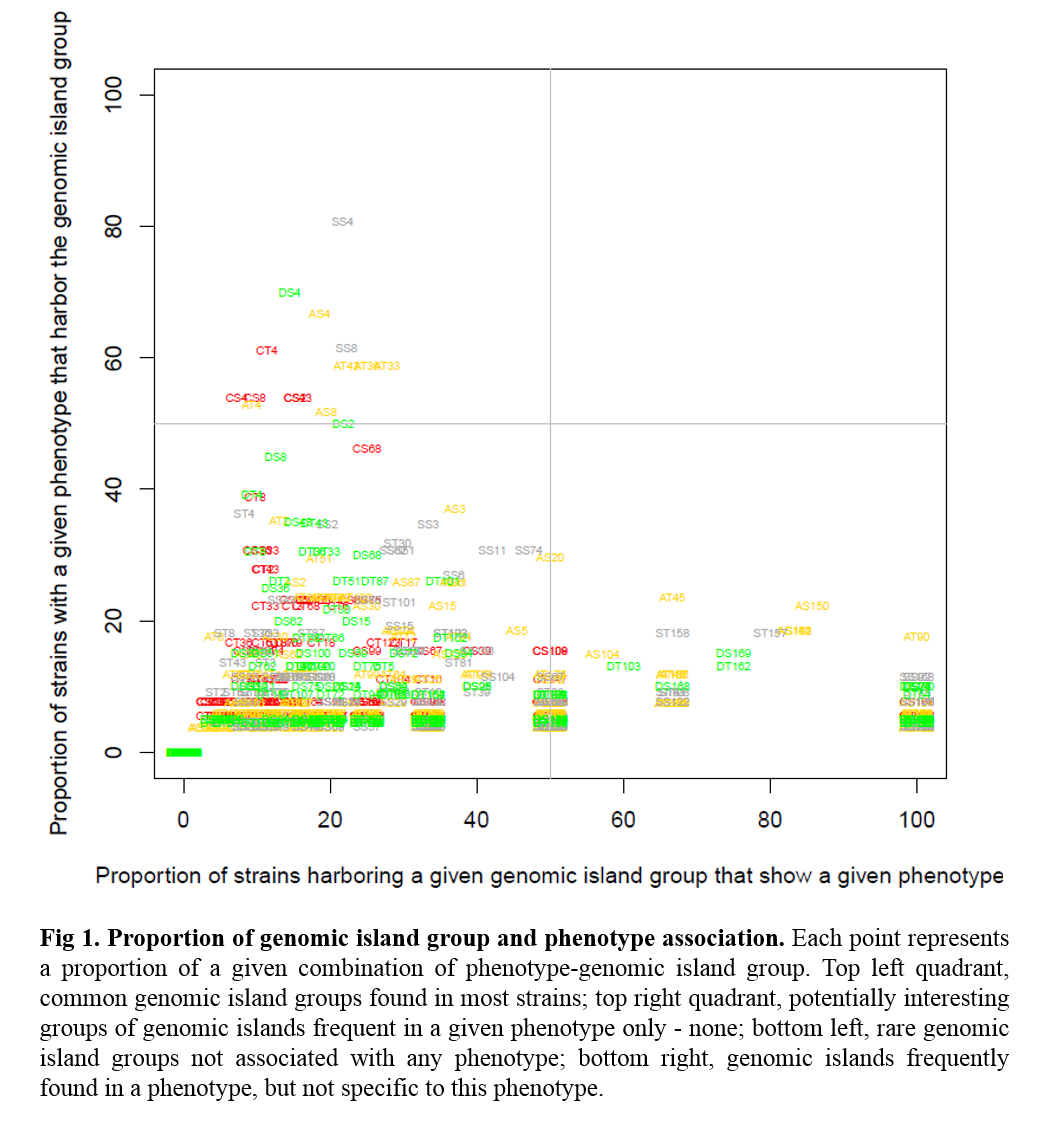


**Figure S1.** Proportion of genomic island group and phenotype association. Each point represents a proportion of a given combination of phenotype-genomic island group. Top left quadrant, common genomic island groups found in most strains; top right quadrant, potentially interesting groups of genomic islands frequent in a given phenotype only - none; bottom left, rare genomic island groups not associated with any phenotype; bottom right, genomic islands frequently found in a phenotype, but not specific to this phenotype.

- 1. **Supplementary Tables**

Files: Table S1-S9.xlsx

**Table S1.** Summary of all *L. monocytogenes* isolates and their associated genetic and phenotypic characteristics. Abbreviations are as follows: AB – Alberta, CA; BC – British Columbia, CA; NS – Nova Scotia, CA; CH – Switzerland; CC – clonal complex; MLST - multilocus sequence type; 3CD – three-codon deletion; std – standardized values; SD – standard deviation, LPD – lag phase duration; µmax – maximum growth rate; Nmax – maximum cell density of absorbance; TRG – time to detectable regrowth; S – sensitive; T- tolerant; SSI-1 – stress survival islet 1, LGI1 – *Listeria* genomic island 1; *Cell surface antigen serotyping was performed.

**Table S2.** SNVs uniquely identified among cold sensitive isolates.

**Table S3.** SNVs uniquely identified among salt sensitive isolates.

**Table S4.** SNVs uniquely identified among acid sensitive isolates.

**Table S5.** SNVs uniquely identified among desiccation sensitive isolates.

**Table S6.** SNVs uniquely identified among cold tolerant isolates.

**Table S7.** SNVs uniquely identified among salt tolerant isolates.

**Table S8.** SNVs uniquely identified among acid tolerant isolates.

**Table S9.** SNVs uniquely identified among desiccation tolerant isolates.
